# Association between admission hyperglycemia and postoperative pneumonia in geriatric patients with hip fractures

**DOI:** 10.1186/s12891-023-06829-5

**Published:** 2023-09-01

**Authors:** Wanyun Tang, Wei Yao, Wei Wang, Qiaomei Lv, Wenbo Ding

**Affiliations:** 1 Department of Orthopedics, Dandong Central Hospital, China Medical University, No. 338 Jinshan Street, Zhenxing District, Dandong, Liaoning Province 118002 P.R. China; 2 Department of Oncology, Dandong Central Hospital, China Medical University, No. 338 Jinshan Street, Zhenxing District, Dandong, Liaoning Province 118002 P.R. China

**Keywords:** Hip fracture, Pneumonia, Hyperglycemia, Risk factor, Geriatric

## Abstract

**Background:**

Admission hyperglycemia is a common phenomenon in the early stages of injury. This study aimed to determine the relationship between admission hyperglycemia and postoperative pneumonia in geriatric patients with hip fractures.

**Methods:**

A total of 600 geriatric patients admitted to Dandong Central Hospital with hip fractures were included. Patients were divided into four groups based on quartiles of admission blood glucose levels: Q1- Q4. Multivariable logistic regression and propensity score-matched analyses were conducted to calculate adjusted odds ratios (ORs) and 95% confidence intervals (CIs) for postoperative pneumonia. Receiver operating characteristic (ROC) curves were used to determine the cut-off value of admission hyperglycemia for predicting postoperative pneumonia.

**Results:**

The incidence of postoperative pneumonia was significantly higher among hyperglycemic patients than those with normal glucose levels (OR = 2.090, 95% CI: 1.135–3.846, *p* = 0.016). Admission hyperglycemia showed moderate predictive power, with an area under the ROC curve of 0.803. Furthermore, propensity score-matched analyses demonstrated that patients in the Q3 (OR = 4.250, 95% CI: 1.361–13.272, *p* = 0.013) and Q4 (OR = 4.667, 95% CI: 1.251–17.405, *p = *0.022) quartiles had a significantly higher risk of postoperative pneumonia compared to patients in the Q1 quartile.

**Conclusions:**

Admission hyperglycemia in elderly hip fracture patients increases the risk of postoperative pneumonia. This biomarker can aid clinical assessment and perioperative management.

**Supplementary Information:**

The online version contains supplementary material available at 10.1186/s12891-023-06829-5.

## Introduction

Hip fracture, defined as a femoral fracture from the hip joint cartilage to a point 5cm below the lesser trochanter, is the most devastating type of fracture [1]. With the global population aging, the worldwide incidence of hip fractures is projected to reach 6.26 million cases by 2050 [2]. Hip fractures significantly impact the quality of life of older adults and are associated with increased mortality and healthcare costs [3, 4]. Early surgical treatment is preferred to facilitate prompt rehabilitation and achieve better prognoses for patients [4, 5]. However, the perioperative period is associated with many complications that significantly increase the mortality rate of elderly patients with hip fractures [6, 7]. Postoperative pneumonia (POP) is a common and severe complication following hip fracture surgery, with an incidence rate ranging from 5.1% to 14.9% [8–11]. POP can result in prolonged hospitalization periods and an increased risk of readmission or death among patients with hip fractures [12, 13].

Hyperglycemia is a common metabolic disorder among patients [14]. Relevant research has shown that hyperglycemia increases the risk of postoperative pneumonia through various mechanisms. Firstly, hyperglycemia suppresses normal immune system function, affecting both cellular and humoral immunity [15]. Secondly, hyperglycemia can lead to bacterial overgrowth and colonization in the respiratory tract and lungs [16]. Finally, hyperglycemia can exacerbate the inflammatory response, leading to tissue damage and activation of immune cells, further contributing to the development of postoperative complications [17]. Strong correlations between intraoperative hyperglycemia and postoperative pneumonia have been confirmed by several studies [18, 19]. Recent literature has also established a link between admission hyperglycemia and POP in patients undergoing liver resection, gastrectomy, and coronary artery bypass grafting [20–23] Compared to surgeries like liver, gastrointestinal, and cardiovascular, hip fracture patients tend to be older with more comorbidities, and have distinct surgical trauma and rehabilitation needs [24]. This may uniquely interact with hyperglycemic levels to produce divergent outcomes from other surgeries. Specifically, common hip fracture surgery complications like thromboembolism and infections directly relate to fracture repair failure, whereas complications of hepatic, gastric, and intestinal surgeries more often involve anastomoses and organ function [20, 22]. Coronary surgery complications primarily involve the cardiovascular system [23]. Given the distinct characteristics of the hip fracture population compared to other cohorts, investigating the relationship between blood glucose and postoperative pneumonia in this group can effectively guide orthopedic clinicians to prevent pneumonia after hip fracture surgery. 

This study investigates the correlation between admission hyperglycemia and postoperative pneumonia incidence in patients with hip fractures. The results will provide scientific evidence to improve pre-operative preparation measures and aid in the effective recovery of geriatric patients with admission hyperglycemia from their hip fractures.

## Methods

### Data sources

A retrospective cohort study was conducted on patients who underwent hip fracture surgery between September 2018 and September 2022 at Dandong Central Hospital, China Medical University, using continuous electronic health records. The Institutional Review Board of Dandong Central Hospital approved the study, and the Ethics Committee granted a consent waiver for the retrospective cohort study. The study followed cohort study guidelines, including a comprehensive collection of demographics, clinical and laboratory data, and utilization of uniform outcome indicators.

### Patient

Inclusion criteria: (1) age 60 or older; (2) confirmed diagnosis of hip fracture through X-ray or CT; (3) confirmation of hip fracture diagnosis during the operation.

Exclusion criteria: (1) absence of surgical intervention; (2) pneumonia confirmed by clinical imaging data before the operation; (3) multiple fractures; (4) incomplete or unavailable data before and after the operation; (5) pathological fractures; and (6) old fractures.

### Blood glucose definitions

The primary exposure was admission hyperglycemia at baseline, defined as the initial non-fasting intravenous blood glucose measurement obtained within the first 24 h following admission. Blood samples were collected from patients during this period. According to the American Diabetes Association's 2022 guidelines [25], we defined normal blood glucose levels as between 4.00–6.09 mmol/L and hyperglycemia as equal to or greater than 6.10 mmol/L. We also categorized patients into four groups based on admission blood glucose levels according to quartiles: Q1 (0–5.3 mmol/L), Q2 (5.3–6.0 mmol/L), Q3 (6.0–6.9 mmol/L), and Q4 (> 6.9 mmol/L). Our hospital has instituted a standardized diagnostic protocol for diabetes in elderly patients with hip fractures, which includes initial screening for hyperglycemia and typical symptoms, confirmatory laboratory testing, detailed review of medical history, and specialist consultations, in order to accurately diagnose diabetes. The flow chart of diabetes diagnosis is shown in Supplementary eFigure 2. Additionally, the specific process of perioperative glucose management is shown in Supplementary eFigure 3 [26].

### Data collection

In this study, we collected a range of indicators, including demographic characteristics, complications, surgery-related factors, and laboratory findings. Demographic data included gender, age, smoking status, and alcohol consumption. Complications assessed were hypertension, diabetes, coronary heart disease, and stroke. Surgical-related factors were hip fracture type, American Society of Anesthesiologists (ASA) grade, operation time, estimated blood loss, and duration of bed rest. In addition to admission blood glucose levels (GLU), laboratory indicators included preoperative measurements of albumin (ALB), hemoglobin (HGB), red blood cells (RBC), white blood cells (WBC), D-dimer, and creatinine (Cr).

These data were extracted from the hospital's electronic medical records. If the patient had multiple measurements of laboratory indicators before surgery, the indicators closest to the admission time were used for analysis by default. The researchers underwent specialized training and made proactive efforts to optimize the data, ensuring its accuracy and effectiveness.

### Outcome

The primary outcome of this study is postoperative pneumonia (POP) in patients with hip fractures. POP is a condition where patients are not diagnosed with pneumonia on preoperative chest X-ray or computed tomography images but develop symptoms, signs, and imaging consistent with pneumonia after surgery and etiological examinations.

According to the guidelines of the American College of Chest Physicians (ACCP) and the European Respiratory Society (ERS), postoperative pneumonia (POP) is defined as a pulmonary infection that occurs within 48–72 h after surgery. The diagnosis of POP requires meeting one or more of the following criteria [27]: (1) new or worsening respiratory symptoms, such as cough and purulent discharge; (2) fever (body temperature > 38°C) or low body temperature (< 36.0°C); (3) lung consolidation or crackles detected during the physical examination; (4) abnormal white blood cell count indicating leukocytosis (> 10 × 109/L) or leukopenia (< 4 × 109/L); (5) identification of relevant pathogens through a culture of microorganisms in sputum or blood samples.

Before commencing the study, four researchers (TWY, YW, WW, and DWB) underwent professional training in identifying pneumonia. Three researchers independently gathered variables, including the identification of pneumonia events. Any discrepancies in variable identification were resolved through discussion with or determination by the Senior Researcher (DWB), as needed.

### Statistical analysis

Descriptive statistics were used to summarize baseline characteristics. Categorical variables were presented as frequency and percentage (n, (%)), and inter-group differences were analyzed using either the chi-square test or Fisher's exact test. Normally distributed continuous variables were expressed as mean ± standard deviation (X ± S), while non-normally distributed variables were expressed as median and interquartile range. We used the Shapiro–Wilk normality test to evaluate the normality status of continuous variables in the baseline data. We compared inter-group differences using both Student t-tests and non-parametric tests. To handle missing data, we utilized multiple imputation, as this method provides less biased estimates compared to other techniques for dealing with missing data.

To address differences in the distribution of groups, we used the nearest neighbor matching algorithm for propensity score matching (PSM) to minimize confounding effects. We conducted a 1:1 PSM between patients with admission hyperglycemia and those without based on their propensity scores, using a caliper width of 0.002. We compared the characteristics of both groups and evaluated balance before and after PSM using the standardized mean difference (SMD) of covariates. When the SMD value is less than 0.5, it suggests minor dissimilarities. Additionally, we performed separate propensity score matching on the three other groups, using the Q1 group as a reference.

A univariate logistic regression and dose–response analysis investigated the relationship between admission hyperglycemia and postoperative pneumonia; the multivariate logistic regression model included covariates with a univariate analysis *P*-value less than 0.10; Odds ratios (ORs), 95% confidence intervals (CIs) and corresponding *P* values indicate the results, with statistical significance set at *P* < 0.05. We established a prediction model for the POP rate based on admission hyperglycemia and evaluated its performance using the Area Under the Curve (AUC). We incorporated multivariable ROC curve analysis and adjusted for covariates to better characterize the association between in-hospital hyperglycemia and the risk of postoperative pneumonia. Finally, we conducted subgroup analyses to investigate the interaction between admission blood glucose and other factors.

SPSS version 26 (SPSS Inc) and R software version 4.0.3 (with Matching and Frailty pack packages developed by the R Foundation for Statistical Computing) are used to conduct all statistical analyses.

## Results

**Fig. 1:**
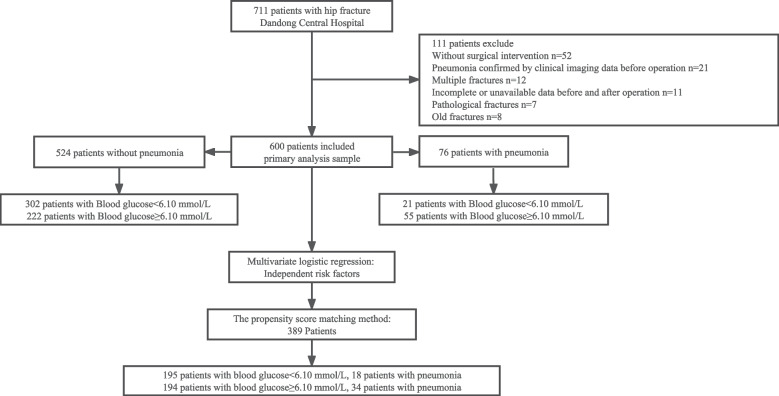
Flow diagram of enrollment

This retrospective study ultimately analyzed 600 patients (Fig. 1). The proportion of patients with diabetes and the specific usage of anti-DM interventions are shown in Supplement eFigure 1. Among the 600 patients in our study, 112 were diagnosed with diabetes. The breakdown of antidiabetic medications used among these diabetic patients is as follows: 14.29% were not on any antidiabetic medication, 32.14% were taking metformin, 24.11% were taking sulfonylureas, 8.93% were taking DPP-4 inhibitors, 7.14% were taking GLP-1 agonists, and 13.39% were using insulin. Among the sample of matched pairs after PSM (*n* = 389; Table 1), those with admission hyperglycemia had higher rates of POP compared to normal blood glucose levels (Table 2). The baseline data characteristics of the blood glucose quartiles after PSM are shown in Table 3. Additional data before PSM are provided in Supplement eTables 1 and 2. These results indicate a significant correlation between admission hyperglycemia and increased risk of POP in this population.

**Table 1:** Patient baseline characteristics before and after PSM by admission blood glucose (≥ 6.10 mmol/L and < 6.10 mmol/L)

Variables	Before PSM			After PSM		
	Glu < 6.10 (*n* = 323)	Glu ≥ 6.10 (*n* = 277)	SMD	Glu < 6.10 (*n* = 195)	Glu ≥ 6.10 (*n* = 194)	SMD
Demographic						
Male gender (n, %)	151(46.70)	95(34.30)	0.255	96(49.20)	74(38.10)	0.224
Age, × year [median (IQR)]	69.00(15.00)	75.00(13.00)	0.269	74.00(17.00)	74.50(14.00)	0.001
Smoking (n, %)	46(14.20)	29(10.50)	0.115	25(12.80)	21(10.80)	0.062
Hypertension (n, %)	116(35.90)	136(49.10)	0.269	75(38.50)	90(46.40)	0.161
Diabetes (n, %)	16(5.00)	96(34.70)	0.802	16(8.20)	16(8.20)	0.002
Cardiovascular disease (n, %)	40(12.10)	44(15.90)	0.100	30(15.40)	26(13.40)	0.056
Stroke (n, %)	61(18.90)	58(20.90)	0.051	39(20.00)	38(19.60)	0.010
Fracture type						
Femoral neck fracture (n, %)	215(66.60)	154(55.60)	0.168	122(62.60)	103(53.10)	0.160
Intertrochanteric fracture (n, %)	79 (24.50)	98(35.40)		52(26.70)	67(34.50)	
Subtrochanteric fracture (n, %)	29(9.00)	25(9.00)		21(10.80)	24(12.40)	
ASA						
III-IV (n, %)	143(44.30)	150(54.20)	0.198	106(54.40)	109(56.20)	0.037
I-II (n, %)	180(55.70)	127(45.80)		89(45.60)	85(43.80)	
Operation surgery						
Intraoperative time, × hour [median (IQR)]	1.50(0.83)	1.50(1.08)	0.139	1.53(1.08)	1.50(1.08)	0.026
Intraoperative blood loss, × ml [median (IQR)]	180.00(145.00)	180.00(200.00)	0.106	200.00(200.00)	196.00(200.00)	0.056
Bedridden time, × day [median (IQR)]	5.00(3.00)	6.00(3.50)	0.133	5.00(3.00)	5.00(3.25)	0.006
Laboratory findings						
ALB, × g/dL [median (IQR)]	39(6.00)	38(6.00)	0.274	38.00(5.00)	38.00(6.00)	0.016
HGB level, × g/L [median (IQR)]	125(25.00)	123(27.5)	0.036	125.00(25.00)	122.50(17.75)	0.071
RBC level, × 10^9/L [median (IQR)]	4.05(0.82)	4.02(0.91)	0.041	4.05(0.88)	4.05(0.94)	0.092
WBC count, × 10^9/L [median (IQR)]	7.80(3.20)	8.70(3.45)	0.095	7.90(3.50)	8.60(3.40)	0.381
D-Dimer, × mg/L [median (IQR)]	3.22(4.48)	3.62(6.24)	0.178	2.98(4.43)	4.24(7.45)	0.269
Cr, × umol/L [median (IQR)]	61.00(24.00)	59.00(27.00)	0.001	64.00(15.00)	59.00(28.00)	0.156

*Abbreviations*: *SMD* standardized mean difference, Used to evaluate the balance before and after PSM, ≥ 0.5 indicates imbalance; *PSM* propensity score matching, *IQR* Interquartile Range, *ASA* the American Society of Anesthesiologists Physical Status Classification System; *ALB* (g/dl, albumin), *HGB* hemoglobin, *RBC* red blood cell, *WBC* White blood cell, *Cr* Creatinine

**Table 2:** Comparison of the incidence of pneumonia after PSM based on admission blood glucose (≥ 6.10 mmol/L vs. < 6.10 mmol/L)

Pneumonia	No. (%)		P*-value
	Glu < 6.10 mmol/L (*n* = 195)	Glu ≥ 6.10 mmol/L (*n* = 194)	
Yes	18(9.2)	34(17.5)	0.016
No	177(90.8)	160(82.5)	

^*^p-value is from Chi-Squared Test to indicate significant differentiation (*P* < 0.05 means significant differentiation)

**Table 3:** Baseline Characteristics of the patients by admission blood glucose (Quartile-based four-category) (mmol/L) after PSM

Characteristics	Total patients (*n* = 389)	Blood glucose Quartile(mmol/L)				*P* for Trend^†^
		Q1(0.00–5.30) (*n* = 77)	Q2(5.30–6.00) (*n* = 118)	Q3(6.00–6.95) (*n* = 115)	Q4(> 6.95) (*n* = 79)	
Demographic						
Male gender (n, %)	170(43.70)	31(40.30)	65(55.10)	44(38.30)	30(38.00)	0.029
Age, × year [median (IQR)]	74.00(15.50)	72.00(15.50)	75.00(18.00)	75.00(16.00)	74.00(12.00)	0.416
Smoking (n, %)	46(11.80)	7(9.10)	18(15.30)	12(10.40)	9(11.40)	0.551
Hypertension (n, %)	165(42.40)	25(32.50)	50(42.40)	43(37.40)	47(59.50)	0.003
Diabetes (n, %)	32(8.20)	2(2.60)	14(11.90)	4(3.50)	12(15.20)	0.003
Cardiovascular disease (n, %)	56(14.40)	12(15.60)	18(15.30)	13(11.30)	13(16.50)	0.725
Stroke (n, %)	77(19.80)	13(16.90)	26(22.00)	17(14.80)	21(26.60)	0.181
Fracture type						
Femoral neck fracture (n, %)	225(57.80)	56(72.70)	66(55.90)	65(56.50)	38(48.10)	0.037
Intertrochanteric fracture (n, %)	119(30.60)	13(16.90)	39(33.10)	37(32.20)	30(38.00)	
Subtrochanteric fracture (n, %)	45(11.60)	8(10.40)	13(11.00)	13(11.30)	11(13.90)	
ASA						
III-IV (n, %)	215(55.30)	40(51.90)	66(55.70)	64(55.70)	45(57.00)	0.927
I-II (n, %)	174(44.70)	37(48.10)	52(44.10)	51(44.30)	87(43.00)	
Operation surgery						
Intraoperative time, × hour [median (IQR)]	1.50(1.08)	1.42(1.05)	1.67(1.08)	1.50(1.00)	1.50(1.08)	0.347
Intraoperative blood loss, × ml [median (IQR)]	200.00(200.00)	200.00(172.00)	200.00(200.00)	180.00(200.00)	200.00(150.00)	0.949
Bedridden time, × day [median (IQR)]	5.00(3.00)	5.00(3.00)	5.00(3.00)	5.00(3.00)	5.00(4.00)	0.900
Laboratory findings						
ALB, × g/dL [median (IQR)]	38.00(5.00)	38.00(4.00)	38.00(5.00)	39.00(5.00)	38.00(6.00)	0.327
HGB level, × g/L [median (IQR)]	123.00(27.00)	125.00(24.50)	125.50(26.00)	123.00(22.00)	118.00(27.00)	0.411
RBC level, 10^9/L [median (IQR)]	4.03(0.87)	4.01(0.90)	4.13(1.26)	4.09(0.87)	3.85 (0.94)	0.157
WBC count, × 10^9/L [median (IQR)]	8.20(3.50)	7.70(3.70)	8.00(3.02)	8.50(3.50)	8.80(3.60)	0.001
D-Dimer, × mg/L [median (IQR)]	3.50(6.09)	2.98(4.45)	3.01(3.56)	4.26(8.19)	4.20(6.27)	0.146
Cr, × μmol/L [median (IQR)]	62.00(18.00)	64.00(29.00)	63.50(26.00)	58.00(24.00)	63.00(20.00)	0.113

^†^*P* values for linear trend for continuous variables are from a generalized linear model, and categorical variables are from an ordinal or logistic regression

There are similar baseline characteristics between normal blood glucose levels (Glu < 6.10) and hyperglycemia (Glu ≥ 6.10) after PSM, indicating that matching has balanced the covariates well between the two groups (Table 1). Patients with admission hyperglycemia (OR 3.563, 95% CI 2.093–6.064, *P* < 0.001) had a significantly higher risk of developing POP than those with normoglycemia. Details of the univariate and multivariate logistic regression analyses for POP are presented in Supplement eTable 3. Even after multiple regression adjustments (Table 4), the results remain robust, showing that patients with admission hyperglycemia (OR 2.644, 95% CI 1.420–4.925, *P* = 0.002) are at increased risk of POP. After PSM adjustment, patients with admission hyperglycemia had a significantly increased risk of POP compared to normoglycemia (OR 2.090, 95% CI 1.135–3.846, *P* = 0.016).

**Table 4:** Comparison of the unadjusted and risk-adjusted POP by different admission blood glucose levels

Outcome	Quartile of Glucose (mmol/L)	Events, n (%)	Unadjusted OR	P* trend 1	Multivariable Regression adjusted OR	*P* trend 2	PSM adjusted OR	*P* trend 3
Postoperative Pneumonia	< 6.10	21 (6.5)	1 [Reference]	< 0.001	1 [Reference]	0.002	1 [Reference]	0.016
	≥ 6.10	55 (19.8)	3.563 (2.093–6.064)		2.644 (1.420–4.925)		2.090 (1.135–3.846)	
	Q1 (0.00–5.30)	5 (3.5)	1 [Reference]		1 [Reference]		1 [Reference]	
	Q2 (5.30–6.00)	16 (8.9)	2.693 (0.962–7.538)	0.059	2.225 (0.774–6.399)	0.138	2.534 (0.868–7.400)	0.089
	Q3 (6.00–6.95)	20 (15.7)	5.159 (1.875–14.192)	0.001	4.994 (1.674–14.901)	0.004	4.250 (1.361–13.272)	0.013
	Q4 (> 6.95)	35 (23.3)	8.400 (3.187–22.140)	< 0.001	5.260 (1.821–15.195)	0.020	4.667 (1.251–17.405)	0.022

The factors of the multivariable regression: Age, Diabetes, Cardiovascular disease, American Society of Anesthesiologists (ASA) Physical Status Classification, Intraoperative time, Intraoperative blood loss, Bedridden time. Albumin, Hemoglobin, Red blood cell, Glu

*CI* confidence interval, *OR* odds ratio, *PSM* propensity scores matching

^*^P for trend

Our analysis showed that higher admission blood glucose levels were associated with an increased risk of postoperative pneumonia (POP). After multivariate regression analysis, compared to patients in group Q1, patients in groups Q2 (OR 2.225, 95% CI 0.774–6.399, *p* = 0.138), Q3 (OR 4.994, 95% CI 1.674–14.901, *p* = 0.004), and Q4 (OR 5.260, 95% CI 1.821–15.195, *p* = 0.020) had a significantly increased risk of POP. After adjustment with propensity score matching (Table 4), compared to patients in Q1, patients in groups Q2 (OR 2.534, 95% CI 0.868–7.400, *p* = 0.089), Q3 (OR 4.250, 95% CI 1.361–13.272, *p* = 0.013), and Q4 (OR 4.667, 95% CI 1.251–17.405, *p* = 0.022) also had a significantly increased risk of POP. This suggests that patients with higher admission blood glucose levels are at higher risk of developing POP. Detailed results for with propensity score matching can be found in Supplementary eTables 4, 5 and 6.

**Fig. 2:**
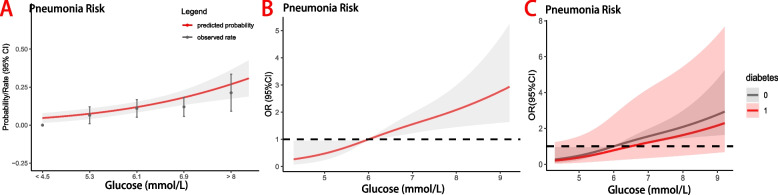
Relationship between admission blood glucose level and POP in patients with hip fracture. **A** Predicted probabilities and the observed rate of POP; **B** Adjusted odd ratios (OR) and corresponding 95% confidence intervals (CI) are presented for each 1.0 mmol/L deviation from the reference value of 6 mmol/L; **C** Adjusted odds ratios (ORs) and their corresponding 95% confidence intervals (CIs) are presented for each 0.1 mmol/L deviation in two groups of diabetic and non-diabetic patients

**Fig. 3:**
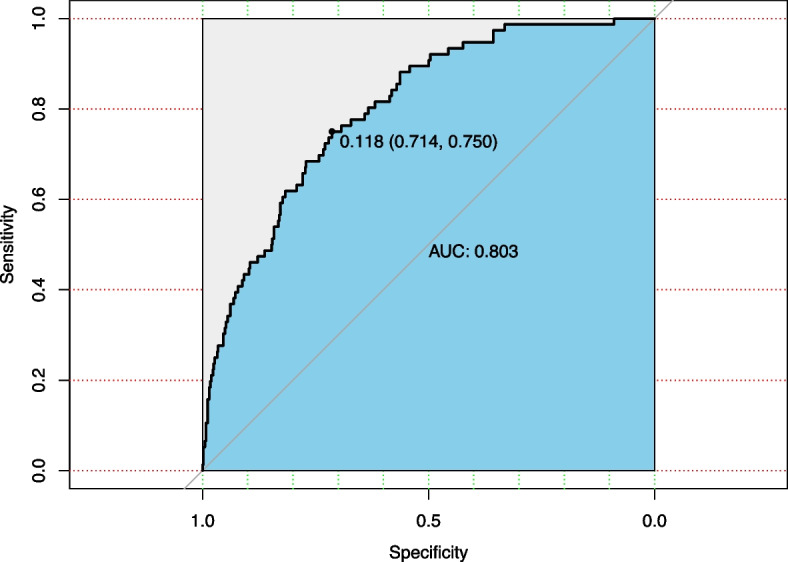
Admission hyperglycemia-based prediction model for postoperative pneumonia rate after hip fracture (AUC)

The dose–response relationship between admission hyperglycemia and POP is illustrated in Fig. 2, indicating a higher incidence of POP associated with admission hyperglycemia. Predicted probability and actual observed values of postoperative pneumonia were obtained based on baseline admission blood glucose levels (Fig. 2A), with the likelihood of POP increasing as baseline blood glucose levels increase. Patients with admission hyperglycemia have a higher risk of POP than those with an admission blood glucose of 6.0 mmol/L(reference). Even when analyzed as a continuous variable, higher blood glucose levels are still associated with an increased likelihood of pneumonia (Fig. 2B). The relationship between admission blood glucose levels and POP in non-diabetic and diabetic patients is presented in Fig. 2C, with non-diabetic patients having a more significant increase in the risk of POP with elevated blood glucose levels compared to diabetic patients. An admission hyperglycemia-based prediction model for POP rate after hip fracture is depicted in Fig. 3, demonstrating a sensitivity of 75.0% and specificity of 71.4%. The area under the ROC curve (AUC) was 0.803, indicating that hyperglycemia before surgery has a strong predictive ability for POP, thus validating our study results. The multivariate ROC curve is shown in Supplement eFigure 4. The AUC values for age, diabetes, bedridden time, and ALB in predicting postoperative pneumonia were 0.6729, 0.5815, 0.6466, and 0.6547, respectively.

**Fig. 4:**
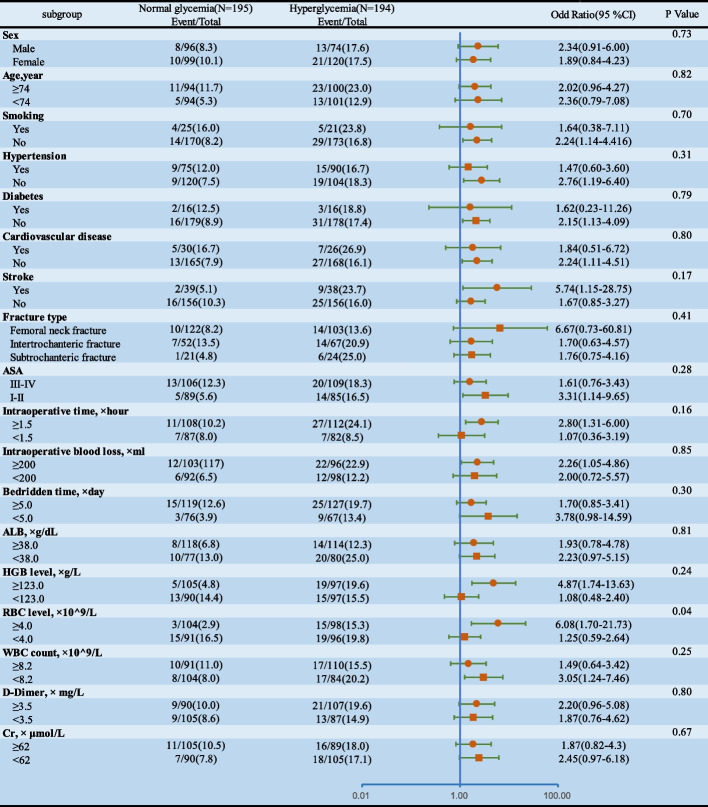
Subgroup analysis of association admission hyperglycemia and POP after propensity score matching

We conducted additional analyses to evaluate the potential interaction between admission hyperglycemia and other variables (Fig. 4). We observed a significant interaction between admission hyperglycemia and RBC (interaction P < 0.05), indicating that the effect of hyperglycemia on the risk of POP may differ depending on RBC levels. However, we found no significant effect modification of the association between admission hyperglycemia and pneumonia risk by the other variables examined.

## Discussion

In this retrospective study, we found that admission hyperglycemia is an independent risk factor for POP in patients undergoing hip fracture surgery. We observed an association between elevated blood glucose at admission and increased risk of POP, with a dose–response effect. Even after conducting logistic regression analysis and propensity score matching to control for various confounding factors, our results remain robust in showing admission hyperglycemia as a risk factor for POP.

Although the specific mechanism by which admission hyperglycemia causes POP in patients is not completely clear, previous research has suggested that it may be through stress-induced hyperglycemia leading to enhanced oxidative stress reactions. This results in the production of harmful molecules such as oxygen and nitrogen free radicals in lung tissue, which can then damage lung tissue structure and function [28]. Numerous studies have shown that elevated blood sugar levels can bind to complement factors and glycosylate immunoglobulins and complement, reducing their function. This ultimately results in a weakened immune response within the body [17, 29, 30]. In addition, stress-induced hyperglycemia can also trigger an inflammatory response, such as increasing the production of inflammatory cytokines (such as TNF-α, IL-1β, and IL-6), which further exacerbate lung tissue inflammation [31, 32]. Furthermore, stress-induced hyperglycemia can suppress immune cell activity, leading to decreased ability of the body to clear bacteria and increasing the risk of POP [17, 33].

Our study results are consistent with previous research on different surgical fields [20–22, 34]. For example, Caroline E. Jones et al. conducted a retrospective observational cohort study of 23,094 surgeries and found that hyperglycemia was an independent risk factor for POP in patients undergoing gastrointestinal surgery [35]. Similarly, Wang Dashuai et al. confirmed in their study that diabetes was an independent risk factor for POP after cardiac surgery [36]. Compared to these studies, our study has the added strength of further adjusting for confounding factors using propensity score matching, thereby minimizing confounding effects and strengthening the evidence for admission hyperglycemia as an independent risk factor for POP.

This study also identified other independent risk factors for POP, including advanced age, diabetes, bed rest time, and hypoalbuminemia. These results are consistent with the findings of other studies. For example, Xiang et al. found in a retrospective study that increasing age, chronic obstructive pulmonary disease, postoperative hypoalbuminemia, and prolonged bed rest time were crucial independent risk factors associated with POP [37]. Wang et al. also confirmed that diabetes increased the risk of POP in patients [36]. In addition, Peng et al. found that prolonged bed rest time was also one of the risk factors for POP in admission hyperglycemia patients [38]. Toshihiro Higashikawa et al. and Wang Y et al. also found that hypoalbuminemia may reduce immune function, leading to tissue edema and metabolic abnormalities, thereby increasing the risk of POP [31, 39]. Junichi Yokota et al. found albumin levels are associated with difficulty swallowing, which in turn is associated with pneumonia, so albumin levels may also be associated with the occurrence of pneumonia [40]. Additionally, a multidisciplinary treatment model combining conventional multidisciplinary therapy and orthogeriatric intervention for reporting on orthogeriatric interventions is an effective and feasible method to reduce various postoperative complications [41]. Some studies also found that hypoalbuminemia may reduce immune function, leading to tissue edema and metabolic abnormalities, thereby increasing the risk of POP [31].

One interesting finding in this retrospective study is worth noting, as shown in Fig. 2b. Compared to diabetic patients, non-diabetic patients had a more significant increase in the risk of POP with an elevated blood glucose level. This result suggests that non-diabetic patients may be more susceptible to the effects of elevated blood glucose, possibly due to a lack of blood glucose control treatment or the presence of other chronic diseases and metabolic abnormalities that increase the risk of pneumonia. However, this finding is merely associative. Further research is needed to investigate the reasons for this phenomenon and how to reduce the risk of pneumonia in non-diabetic patients after surgery.

In the interaction analysis, patients with RBC ≥ 4.0 × 10^9/L showed a significant association between admission blood glucose level and postoperative pneumonia, while there was no association for patients with RBC < 4.0 × 10^9/L (*P* = 0.04). According to relevant literature, false positives may occur when analyzing multiple subgroups [41], so this observed interaction between hyperglycemia and RBC levels requires further investigation.

The retrospective study has several strengths. Firstly, there has yet to be any prior research on the association between admission hyperglycemia and POP in hip fracture patients. Secondly, propensity score matching was utilized to mitigate the influence of diverse confounding factors. Thirdly, we conducted a dose–response analysis to visualize the relationship between admission hyperglycemia and POP. Finally, in this study, we used the area under the ROC curve (AUC) to evaluate the ability of admission hyperglycemia to predict POP effectively.

However, several limitations in this study need further improvement and exploration. Firstly, our study has certain constraints due to its retrospective design. Secondly, the sample size of POP is small and only from a single institution, so the applicability to a broader population remains to be verified. Thirdly, although standardized diagnostic criteria were established before the study, clinical diagnosis has a certain degree of subjectivity and variability. The actual incidence of POP may be underestimated or overestimated. Additionally, due to the retrospective nature of this study, we were unable to systematically account for potential confounders like cognitive impairment and COPD, which could attenuate the observed associations with postoperative pneumonia risk according to prior studies. Finally, we did not strictly distinguish whether hyperglycemia was caused by trauma-induced acute hyperglycemia or diabetes. We did not conduct a subgroup analysis of diabetes drugs. These two points may have led to some confounding effects of diabetes in our results.

## Conclusions

We found that admission hyperglycemia has a dose–response association with postoperative pneumonia in geriatric patients with hip fractures. An increase in admission blood glucose was associated with a higher risk of postoperative pneumonia. This biomarker could provide important information for the prevention and preoperative optimization of postoperative pneumonia in hip fracture patients and may help reduce perioperative mortality and complications in these patients.

### Supplementary Information

**Additional file 1: eTable 1.** Baseline characteristics of the patients by admission blood glucose (Quartile-based four-category) (mmol/L) before PSM. **eTable 2.** Comparison of the incidence of pneumonia before PSM based on glucose level (≥6.10 mmol/L vs <6.10 mmol/L). **eTable 3.** Univariate and multivariate regression analyses of risk factors for POP. **eTable 4.** Patient characteristics before and after PSM by glucose level (Q1 [0.00-5.30] vs. Q2[5.30-6.00] mmol/L). **eTable 5.** Patient characteristics before and after PSM by glucose level (Q1 [0.00-5.30] vs. Q3[6.00-6.95] mmol/L). **eTable 6.** Patient characteristics before and after PSM by glucose level (Q1 [0.00-5.30] vs. Q4[≥6.95] mmol/L). **eFigure 1.** The proportion of patients with diabetes and the specific usage of anti-DM interventions. **eFigure 2.** Flow chart of diabetes diagnosis. **eFigure 3.** The specific process of perioperative glucose management. **eFigure 4.** The multivariate ROC analysis of risk factors for POP.

## Data Availability

All the data used and analyzed during the current study are available from the corresponding author upon reasonable request.
