# Supplementary material for: Association between admission hyperglycemia and postoperative pneumonia in geriatric patients with hip fractures
Source: BMC Musculoskelet Disord. 2023 Sep 1;24:700. doi: 10.1186/s12891-023-06829-5 (PMC10472715; doi:10.1186/s12891-023-06829-5)

**Supplement**

# eTable 1 Baseline Characteristics of the Patients by Admission Blood Glucose (Quartile-based four-category) (mmol/L) before PSM.

| Characteristics | Total patients  (n = 600) | Blood glucose Quartile(mmol/L) | | | | P for Trend† |
| --- | --- | --- | --- | --- | --- | --- |
|  |  | 0.00-5.30  (n = 143) | 5.30–6.00  (n = 180) | 6.00-6.95  (n = 127) | > 6.95  (n = 150) |  |
| Demographic |  |  |  |  |  |  |
| Male gender (n, %) | 246(41.0) | 68(47.6) | 83(46.1) | 46(36.0) | 49(32.7) | 0.019 |
| Age, ×year [median (IQR)] | 72.0(14.0) | 68.00(15.0) | 70.5(17.8) | 75.00(16.00) | 75.00(12.00) | <0.001 |
| Smoking (n, %) | 75(12.5) | 19(13.3) | 30(16.7) | 20(15.7) | 23(15.3) | 0.867 |
| Hypertension (n, %) | 252(42.0) | 46(32.2) | 70(38.9) | 49(38.6) | 87(58.0) | <0.001 |
| Diabetes (n, %) | 112(18.7) | 3(2.1) | 14(7.8) | 13(10.2) | 68(54.7) | <0.001 |
| Cardiovascular disease (n, %) | 84(14.0) | 18(12.6) | 22(11.7) | 15(11.8) | 29(19.3) | 0.283 |
| Stroke (n, %) | 119(19.8) | 26(18.2) | 35(19.4) | 17(13.4) | 41(27.3) | 0.031 |
| Fracture type |  |  |  |  |  |  |
| Femoral neck fracture (n, %) | 369(61.5) | 103(72.0) | 112(62.2) | 71(55.9) | 83(55.30) | 0.026 |
| Intertrochanteric fracture (n, %) | 177(29.5) | 26(18.2) | 53(29.4) | 43(33.9) | 55(36.7) |  |
| Subtrochanteric fracture (n, %) | 54(9.0) | 14(9.8) | 15(8.3) | 13(10.2) | 12(8.00) |  |
| ASA |  |  |  |  |  |  |
| Ⅲ-Ⅳ (n, %) | 293(48.8) | 59(41.3) | 84 (46.7) | 70(55.1) | 80(53.3) | 0.077 |
| Ⅰ-Ⅱ (n, %) | 307(52.0) | 84(58.7) | 96(53.3) | 57(44.9) | 70(46.7) |  |
| Operation surgery |  |  |  |  |  |  |
| Intraoperative time, ×hour [median (IQR)] | 1.5(0.9) | 1.5(0.8) | 1.5(0.8) | 1.5(1.8) | 1.5(0.8) | 0.334 |
| Intraoperative blood loss, ×ml [median (IQR)] | 180.0(158.8) | 200.0(150.0) | 150.0(135.0) | 180.0(200.0) | 170.0(131.5) | 0.578 |
| Bedridden time, ×day [median (IQR)] | 5.0(3.0) | 5.0(3.0) | 6.0(4.0) | 5.00(3.0) | 5.5(3.3) | 0.306 |
| Laboratory findings |  |  |  |  |  |  |
| ALB, ×g/dL [median (IQR)] | 39.0(5.0) | 39.0(6.0) | 39.0(3.3) | 39.00(5.00) | 38.0(6.0) | 0.031 |
| HGB level, ×g/L [median (IQR)] | 123.0(26.0) | 123.0(27.0) | 125.0(22.0) | 123.0(25.0) | 122.5(29.0) | 0.951 |
| RBC level, ×10^9/L [median (IQR)] | 4.0(1.0) | 4.05(1.0) | 4.1(0.7) | 4.1(0.9) | 4.0(1.0) | 0.871 |
| WBC count, ×10^9/L [median (IQR)] | 8.2(3.3) | 7.7(3.6) | 8.0(2.9) | 8.5(3.5) | 8.8(3.6) | 0.469 |
| D-Dimer，×mg/L [median (IQR)] | 3.4(5.0) | 3.0(4.3) | 3.5(5.0) | 3.7(8.0) | 3.6(4.9) | 0.141 |
| Cr, × μmol /L [median (IQR)] | 60.0(24.8) | 63.0(23.0) | 59.5(24.0) | 58.0(24.0) | 61.5(29.3) | 0.098 |

# † P values for linear trend for continuous variables are from a generalized linear model, and categorical variables are from an ordinal or logistic regressioneTable 2 Comparison of the Incidence of Pneumonia before PSM based on Glucose Level (≥6.10 mmol/L vs <6.10 mmol/L)

| Pneumonia | No. (%) | | p-value* |
| --- | --- | --- | --- |
|  | Blood glucose<6.10 mmol/L  (n=323) | Blood glucose≥6.10 mmol/L  (n=277) |  |
| Yes | 21(6.5) | 55(19.8) | <0.001 |
| No | 302(93.5) | 222(80.2) |  |

*p-value is from Chi-Squared Test to indicate significant differentiation (P<0.05 indicates significant differentiation)

# eTable 3 Univariate and Multivariate Regression Analyses of Risk Factors for POP

| Variables | Univariate | | | Multivariate | | |
| --- | --- | --- | --- | --- | --- | --- |
|  | OR | 95%CI | P-value | OR | 95%CI | P-value |
| Demographic |  |  |  |  |  |  |
| Male gender (n, %) | 0.873 | 0.532-1.431 | 0.590 |  |  |  |
| Age (years, mean ± SD) | 1.072 | 1.042-1.102 | <0.001 | 1.046 | 1.010-1.083 | 0.012 |
| Smoking (n, %) | 1.372 | 0.702-2.682 | 0.355 |  |  |  |
| Hypertension (n, %) | 1.208 | 0.745-1.958 | 0.444 |  |  |  |
| Diabetes (n, %) | 2.287 | 1.339-3.905 | 0.002 | 1.994 | 1.048-3.793 | 0.035 |
| Cardiovascular disease (n, %) | 1.727 | 0.974-3.283 | 0.061 | 1.301 | 0.645-2.625 | 0.462 |
| Stroke (n, %) | 0.993 | 0.543-1.817 | 0.982 |  |  |  |
| Fracture type | 0.795 | 0.561-1.127 | 0.198 |  |  |  |
| ASA | 2.224 | 1.343-3.681 | 0.002 | 1.361 | 0.752-2.464 | 0.309 |
| Operation surgery |  |  |  |  |  |  |
| Intraoperative time (hour, mean ± SD) | 1.528 | 1.178-1.981 | 0.001 | 1.364 | 0.967-1.924 | 0.077 |
| Intraoperative blood loss (ml, mean ± SD) | 1.001 | 1.000-1.003 | 0.015 | 1.001 | 0.999-1.002 | 0.490 |
| Bedridden time (day, mean ± SD) | 1.12 | 1.053-1.191 | <0.001 | 1.077 | 1.000-1.161 | 0.050 |
| Laboratory indicators |  |  |  |  |  |  |
| ALB, g/Dl | 0.875 | 0.828-0.923 | <0.001 | 0.839 | 0.784-0.897 | <0.001 |
| HGB level (g/L, mean ± SD) | 0.981 | 0.969-0.993 | 0.002 | 1.011 | 0.986-1.036 | 0.406 |
| RBC level (n*109/L, mean ± SD) | 0.506 | 0.347-0.737 | <0.001 | 0.877 | 0.418-1.842 | 0.729 |
| WBC count (n*109/L, mean ± SD) | 1.017 | 0.994-1.041 | 0.147 |  |  |  |
| D-Dimer (ng/ml, mean ± SD) | 1.027 | 0.991-1.064 | 0.142 |  |  |  |
| Cr (μmol /L, mean ± SD) | 1.00 | 0.996-1.004 | 0.898 |  |  |  |
| Glu (≥6.10 mmol/) | 3.563 | 2.093-6.064 | <0.001 | 2.644 | 1.420-4.925 | 0.002 |

Note: Data are presented as mean ± standard deviation or number (%)

Abbreviations: SMD, standardized mean difference, Used to evaluate the balance before and after PSM, ≥ 0.5 indicates imbalance; PSM: propensity score matching; IQR: Interquartile Range; ASA: the American Society of Anesthesiologists Physical Status Classification System; ALB（g/Dl）， albumin; HGB, hemoglobin; RBC, red blood cell; WBC: White blood cell; Cr, Creatinine

# eTable 4 Patient Characteristics Before and After PSM by Glucose Level (Q1 [0.00-5.30] vs. Q2[5.30-6.00] mmol/L).

| Variables | Before PSM | | | After PSM | | |
| --- | --- | --- | --- | --- | --- | --- |
|  | Q1 [0.00-5.30] (n=143) | Q2[5.30-6.00]  (n=180) | SMD | Q1 [0.00-5.30] (n=139) | Q2[5.30-6.00] (n=139) | SMD |
| Demographic |  |  |  |  |  |  |
| Male gender (n, %) | 68(47.6) | 83(46.1) | 0.029 | 66(47.5) | 65(46.8) | 0.014 |
| Age, ×year [median (IQR)] | 68.0(15.0) | 70.5(17.8) | 0.243 | 68.0(14.0) | 69.0(16.0) | 0.032 |
| Smoking (n, %) | 19(13.3) | 30(16.7) | 0.049 | 18(12.9) | 23(16.5) | 0.101 |
| Hypertension (n, %) | 46(32.2) | 70(38.9) | 0.140 | 46(33.1) | 52(37.4) | 0.090 |
| Diabetes (n, %) | 2(1.4) | 14(7.8) | 0.308 | 2(1.4) | 2(1.4) | <0.001 |
| Cardiovascular disease (n, %) | 18(12.60) | 22(11.7) | 0.011 | 18(12.9) | 16(11.5) | 0.044 |
| Stroke (n, %) | 26(18.2) | 35(19.4) | 0.032 | 26(18.7) | 24(17.3) | 0.037 |
| Fracture type |  |  |  |  |  |  |
| Femoral neck fracture (n, %) | 103(72.0) | 112(62.2) | 0.128 | 101(72.7) | 86(61.9) | 0.145 |
| Intertrochanteric fracture (n, %) | 26(18.2) | 53(29.4) |  | 25(18.0) | 42(30.2) |  |
| Subtrochanteric fracture (n, %) | 14(9.8) | 15(8.3) |  | 13(9.4) | 11(7.9) |  |
| ASA |  |  |  |  |  |  |
| Ⅲ-Ⅳ (n, %) | 59(41.3) | 84 (46.7) | 0.109 | 59(42.4) | 59(42.4) | <0.001 |
| Ⅰ-Ⅱ (n, %) | 84(58.7) | 96(53.3) |  | 80(57.6) | 80(57.6) |  |
| Operation surgery |  |  |  |  |  |  |
| Intraoperative time, ×hour [median (IQR)] | 1.5(0.8) | 1.5(0.8) | 0.077 | 1.5(0.8) | 1.50(0.8) | 0.100 |
| Intraoperative blood loss, ×ml [median (IQR)] | 200.0(150.0) | 150.0(135.0) | 0.017 | 200.0(150.0) | 150.0(145.0) | 0.056 |
| Bedridden time, ×day [median (IQR)] | 5.00(3.0) | 6.00(4.0) | 0.096 | 5.0(4.0) | 5.0(3.0) | 0.025 |
| Laboratory findings |  |  |  |  |  |  |
| ALB, ×g/Dl [median (IQR)] | 39.0(6.0) | 39.0(3.3) | 0.061 | 39.0(7.0) | 39.0(5.0) | 0.029 |
| HGB level, ×g/L [median (IQR)] | 123.0(27.0) | 125.0(22.0) | 0.034 | 125.0(27.0) | 126.0(23.0) | 0.074 |
| RBC level, ×10^9/L [median (IQR)] | 4.1(1.0) | 4.1(0.7) | 0.059 | 4.1(1.0) | 4.1(0.8) | 0.097 |
| WBC count, ×10^9/L [median (IQR)] | 7.7(3.6) | 8.0(2.9) | 0.052 | 7.7(3.6) | 8.0(2.8) | 0.079 |
| D-Dimer，×mg/L [median (IQR)] | 3.0(4.3) | 3.5(5.0) | 0.072 | 3.0(4.3) | 3.5(5.2) | 0.108 |
| Cr, × μmol/L [median (IQR)] | 63.0 (23.0) | 59.5(24.0) | 0.227 | 64.0(24.0) | 58.0(21.0) | 0.271 |

Note: Data are presented as mean ± standard deviation or number (%)

Abbreviations: SMD, standardized mean difference, used to evaluate the balance before and after PSM, ≥ 0.5 indicates imbalance; PSM: propensity score matching; IQR: Interquartile Range; ASA: the American Society of Anesthesiologists Physical Status Classification System; ALB (g/Dl), albumin; HGB, hemoglobin; RBC, red blood cell; WBC: White blood cell; Cr, Creatinine

# eTable 5 Patient Characteristics Before and After PSM by Glucose Level (Q1 [0.00-5.30] vs. Q3[6.00-6.95] mmol/L).

| Variables | Before PSM | | | After PSM | | |
| --- | --- | --- | --- | --- | --- | --- |
|  | Q1 [0.00-5.30]  (n=143) | Q3[6.00-6.95]  (n=180) | SMD | Q1 [0.00-5.30]  (n=106) | Q3[6.00-6.95]  (n=105) | SMD |
| Demographic |  |  |  |  |  |  |
| Male gender (n, %) | 68(47.6) | 46(36.2) | 0.230 | 46(43.4) | 42(40.0) | 0.069 |
| Age, ×year [median (IQR)] | 68.0(15.0) | 75.0(16.0) | 0.356 | 72.5(13.0) | 74.0(15.0) | 0.034 |
| Smoking (n, %) | 19(13.3) | 14(11.0) | 0.069 | 16(15.1) | 12(11.4) | 0.108 |
| Hypertension (n, %) | 46(32.2) | 49(38.6) | 0.134 | 39(36.8) | 35(33.3) | 0.072 |
| Diabetes (n, %) | 2(1.4) | 14(11.0) | 0.405 | 2(1.9) | 2(1.9) | 0.001 |
| Cardiovascular disease (n, %) | 18(12.60) | 15(11.8) | 0.024 | 15(14.2) | 10(9.5) | 0.143 |
| Stroke (n, %) | 26(18.2) | 17(13.4) | 0.131 | 23(21.7) | 14(13.3) | 0.220 |
| Fracture type |  |  |  |  |  |  |
| Femoral neck fracture (n, %) | 103(72.0) | 71(55.9) | 0.248 | 73(68.9) | 61(58.1) | 0.205 |
| Intertrochanteric fracture (n, %) | 26(18.2) | 43(33.9) |  | 24(12.6) | 32(30.5) |  |
| Subtrochanteric fracture (n, %) | 14(9.8) | 13(10.2) |  | 9(8.5) | 12(11.4) |  |
| ASA |  |  |  |  |  |  |
| Ⅲ-Ⅳ (n, %) | 59(41.3) | 70(55.1) | 0.279 | 50(47.2) | 57(54.3) | 0.142 |
| Ⅰ-Ⅱ (n, %) | 84(58.7) | 57(44.9) |  | 56(52.8) | 48(45.7) |  |
| Operation surgery |  |  |  |  |  |  |
| Intraoperative time, ×hour [median (IQR)] | 1.5(0.8) | 1.5(1.1) | 0.191 | 1.4(0.8) | 1.6(1.1) | 0.229 |
| Intraoperative blood loss, ×ml [median (IQR)] | 200.0(150.0) | 180.0(200.0) | 0.074 | 200.0(200.0) | 170.0(200.0) | 0.001 |
| Bedridden time, ×day [median (IQR)] | 5.0(3.0) | 6.0(4.0) | 0.048 | 5.0(4.3) | 5.0(3.5) | 0.011 |
| Laboratory findings |  |  |  |  |  |  |
| ALB, ×g/dL [median (IQR)] | 39.0(6.0) | 39.0(5.0) | 0.207 | 38.0(6.0) | 39.0(5.0) | 0.041 |
| HGB level, ×g/L [median (IQR)] | 123.0(27.0) | 123.0(25.0) | <0.001 | 121.0(27.3) | 124.0(23.5) | 0.135 |
| RBC level, ×10^9/L [median (IQR)] | 4.1(1.0) | 4.1(0.9) | 0.037 | 4.0(10.9) | 4.1(0.8) | 0.222 |
| WBC count, ×10^9/L [median (IQR)] | 7.7(3.6) | 8.5(3.5) | 0.077 | 7.4(3.7) | 8.6(3.3) | 0.056 |
| D-Dimer, ×mg/L [median (IQR)] | 3.0(4.3) | 3.7(8.0) | 0.265 | 3.0(4.3) | 4.2(8.3) | 0.310 |
| Cr, × μmol/L [median (IQR)] | 63.0 (23.0) | 58.0(24.0) | 0.244 | 62.5(25.5) | 59.0(26.5) | 0.232 |

Note: Data are presented as mean ± standard deviation or number (%)

Abbreviations: SMD, standardized mean difference, used to evaluate the balance before and after PSM, ≥ 0.5 indicates imbalance; PSM: propensity score matching; IQR: Interquartile Range; ASA: the American Society of Anesthesiologists Physical Status Classification System; ALB (g/Dl), albumin; HGB, hemoglobin; RBC, red blood cell; WBC: White blood cell; Cr, Creatinine

**eTable 6 Patient Characteristics Before and After PSM by Glucose Level (Q1 [0.00-5.30] vs. Q4[≥6.95] mmol/L).**

| Variables | Before PSM | | | After PSM | | |
| --- | --- | --- | --- | --- | --- | --- |
|  | Q1 [0.00-5.30]  (n=143) | Q4[≥6.95]  (n=150) | SMD | Q1 [0.00-5.30]  (n=66) | Q4[≥6.95]  (n=66) | SMD |
| Demographic |  |  |  |  |  |  |
| Male gender (n, %) | 68(47.6) | 49(32.7) | 0.306 | 31(47.0) | 26(49.4) | 0.152 |
| Age, ×year [median (IQR)] | 68.0(15.0) | 75(16.0) | 0.491 | 68.0(15.0) | 75.0(16.0) | 0.025 |
| Smoking (n, %) | 19(13.3) | 15(10.0) | 0.102 | 6(9.1) | 8(12.1) | 0.098 |
| Hypertension (n, %) | 46(32.2) | 87(58.0) | 0.536 | 26(39.4) | 38(57.6) | 0.367 |
| Diabetes (n, %) | 2(1.4) | 82(54.7) | 1.468 | 2(3.0) | 2(3.0) | <0.001 |
| Cardiovascular disease (n, %) | 18(12.6) | 29(19.3) | 0.184 | 6(9.1) | 11(16.7) | 0.226 |
| Stroke (n, %) | 26(18.2) | 41(27.3) | 0.219 | 13(19.7) | 16(24.2) | 0.109 |
| Fracture type |  |  |  |  |  |  |
| Femoral neck fracture (n, %) | 103(72.0) | 83(55.3) | 0.229 | 47(71.2) | 31(47.0) | 0.407 |
| Intertrochanteric fracture (n, %) | 26(18.2) | 55(36.7) |  | 13(19.7) | 27(40.9) |  |
| Subtrochanteric fracture (n, %) | 14(9.8) | 12(8.0) |  | 6(9.1) | 8(12.1) |  |
| ASA |  |  |  |  |  |  |
| Ⅲ-Ⅳ (n, %) | 59(41.3) | 80(53.3) | 0.243 | 31(47.0) | 38(57.6) | 0.212 |
| Ⅰ-Ⅱ (n, %) | 84(58.7) | 70(46.7) |  | 35(53.0) | 28(42.4) |  |
| Operation surgery |  |  |  |  |  |  |
| Intraoperative time, hour [median (IQR)] | 1.5(0.9) | 1.5(1.1) | 0.171 | 1.5(0.9) | 1.5(1.1) | 0.259 |
| Intraoperative blood loss, ×ml [median (IQR)] | 200.0(150.0) | 180.00(200.0) | 0.116 | 200.0(150.0) | 180.0(200.0) | 0.031 |
| Bedridden time, ×day [median (IQR)] | 5.00(3.0) | 6.00(4.0) | 0.097 | 5.0(3.0) | 6.0(4.0) | 0.059 |
| Laboratory findings |  |  |  |  |  |  |
| ALB, ×g/dL [median (IQR)] | 39.00(6.0) | 39.00(5.0) | 0.365 | 39.0(6.0) | 39.0(5.0) | 0.068 |
| HGB level, ×g/L [median (IQR)] | 123.0(27.0) | 123.0(25.0) | 0.032 | 123.0(27.0) | 123.0(25.0) | 0.216 |
| RBC level, ×10^9/L [median (IQR)] | 4.1(1.0) | 4.1(0.9) | 0.073 | 4.1(1.0) | 4.1(0.9) | 0.289 |
| WBC count, ×10^9/L [median (IQR)] | 7.7(3.6) | 8.5(3.5) | 0.190 | 7.7(3.6) | 8.5(3.5) | 0.036 |
| D-Dimer, ×mg/L [median (IQR)] | 3.0(4.3) | 3.7(8.0) | 0.175 | 3.0(4.3) | 3.7(8.0) | 0.478 |
| Cr, ×μmol/L [median (IQR)] | 63.0 (23.0) | 58.0(24.0) | 0.021 | 63.0(23.0) | 58.0(24.0) | 0.104 |

Note: Data are presented as mean ± standard deviation or number (%)

Abbreviations: SMD, standardized mean difference, Used to evaluate the balance before and after PSM, ≥ 0.5 indicates imbalance; PSM: propensity score matching; IQR: Interquartile Range; ASA: the American Society of Anesthesiologists Physical Status Classification System; ALB (g/Dl), albumin; HGB, hemoglobin; RBC, red blood cell; WBC: White blood cell; Cr, Creatinine

**eFigure 1:** The Proportion of Patients with Diabetes and the Specific Usage of Anti-DM Interventions


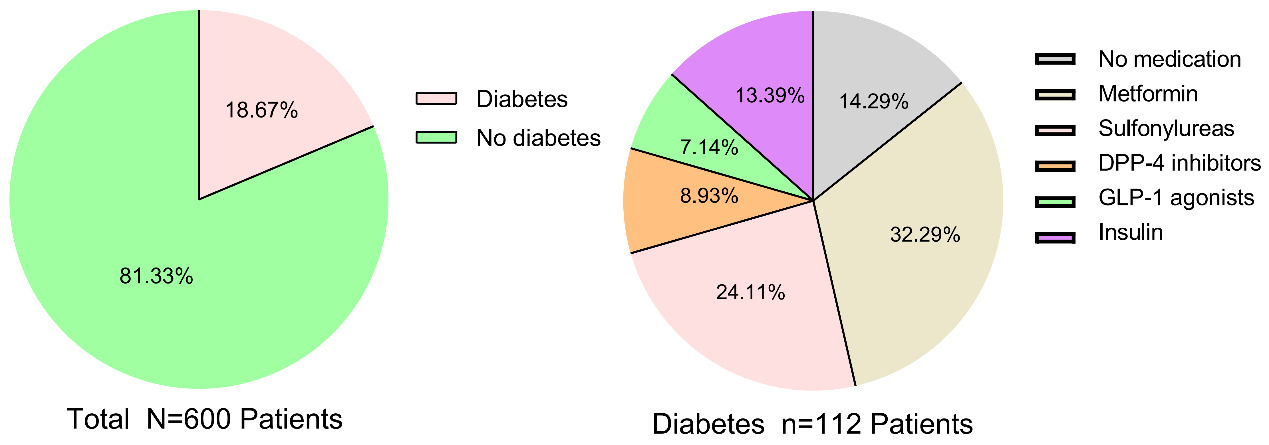


**eFigure 2:**Flow Chart of Diabetes Diagnosis


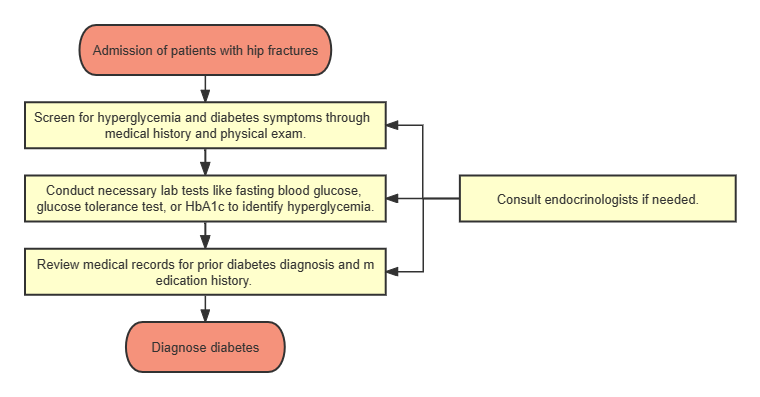


**eFigure 3:** The Specific Process of Perioperative Glucose Management **
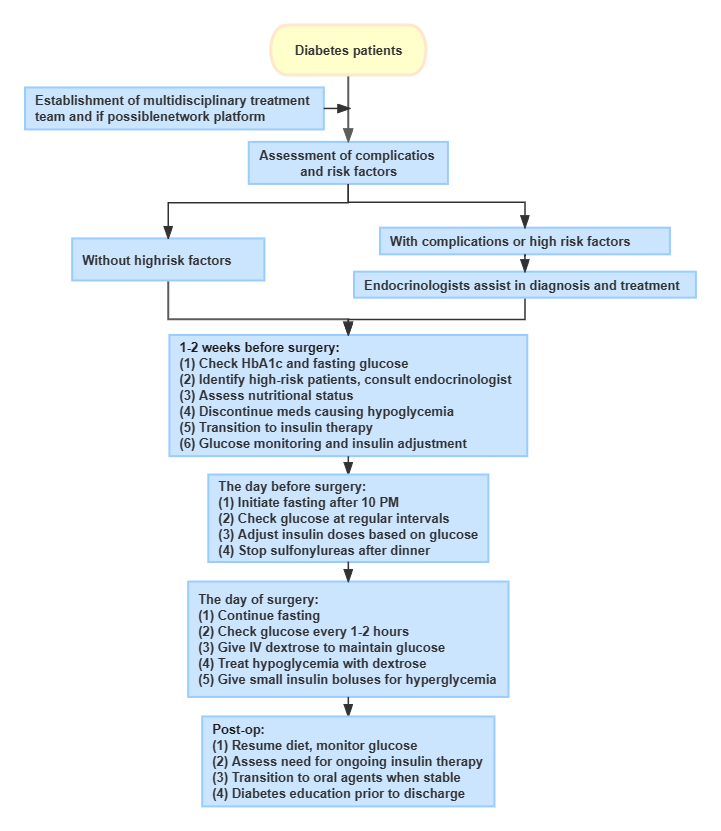
**

**eFigure 4:** The Multivariate ROC Analysis of Risk Factors for POP


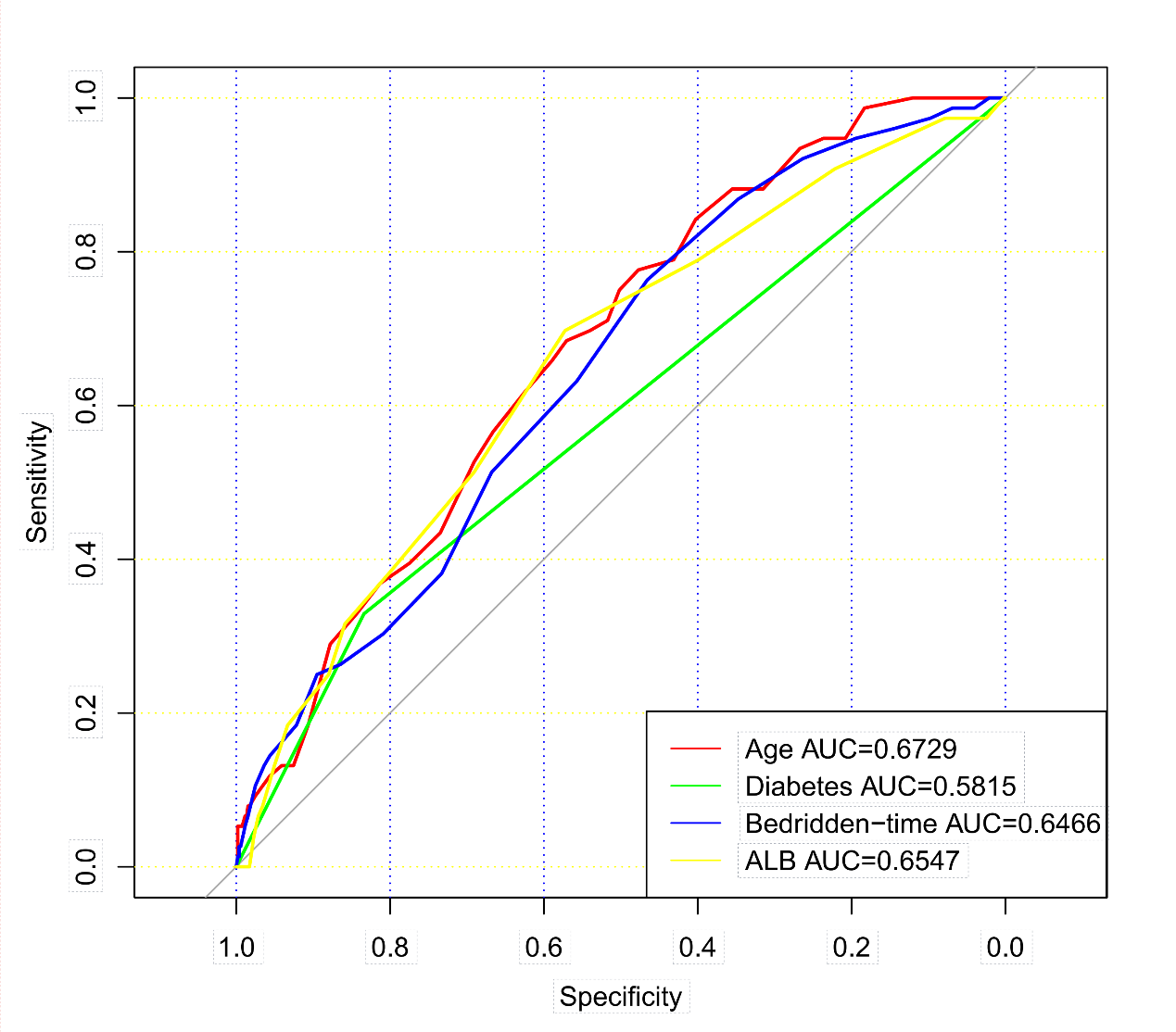

Supplement: Supplementary file 1 — Additional file 1: eTable 1. Baseline characteristics of the patients by admission blood glucose (Quartile-based four-category) (mmol/L) before PSM. eTable 2. Comparison of the incidence of pneumonia before PSM based on glucose level (≥6.10 mmol/L vs <6.10 mmol/L). eTable 3. Univariate and multivariate regression analyses of risk factors for POP. eTable 4. Patient characteristics before and after PSM by glucose level (Q1 [0.00-5.30] vs. Q2[5.30-6.00] mmol/L). eTable 5. Patient characteristics before and after PSM by glucose level (Q1 [0.00-5.30] vs. Q3[6.00-6.95] mmol/L). eTable 6. Patient characteristics before and after PSM by glucose level (Q1 [0.00-5.30] vs. Q4[≥6.95] mmol/L). eFigure 1. The proportion of patients with diabetes and the specific usage of anti-DM interventions. eFigure 2. Flow chart of diabetes diagnosis. eFigure 3. The specific process of perioperative glucose management. eFigure 4. The multivariate ROC analysis of risk factors for POP. [file 12891_2023_6829_MOESM1_ESM.docx]
